# The Lipari-Szabo Model-Free Analysis as a Method for Study of Molecular Motion in Solid State Heteronuclear Systems Using NMR Off-Resonance

**DOI:** 10.1007/s00723-016-0785-5

**Published:** 2016-04-28

**Authors:** A. Woźniak-Braszak, K. Jurga, M. Baranowski

**Affiliations:** High Pressure Physics Division, Faculty of Physics, Adam Mickiewicz University, Umultowska 85, 61-614 Poznan, Poland

## Abstract

In the present work, a new method for measuring motional parameters using the off-resonance technique was described. The Lipari-Szabo model-free formalism was used to analyze molecular dynamics in a heteronuclear system [1, 2]. Cross-relaxation solid state nuclear magnetic resonance off-resonance experiments were performed on a homebuilt pulse spectrometer operating at the frequency of 30.2 MHz for protons at temperature 173 K. The proton spins were spin-locked in the effective field $\vec{B}_{e}$ while ^19^F spins were continuously saturated for a long time. It was possible to carry out these experiments because a uniquely designed probe was able to produce three slightly differing frequencies on and off-resonance for protons and the frequency of 28.411 MHz for fluorine [3–6]. The off-resonance frequencies can be changed within the range of 30.2–30.6 MHz.

## Introduction

Solid state nuclear magnetic resonance (NMR) off-resonance measurements provide valuable information about molecular dynamics in a heteronuclear system [7–10]. So far only a few authors have presented the results of study of the cross-relaxation in the heteronuclear systems in the solid state [7, 10–12]. Complex analysis of these systems and apparatus requirements pose problems which are difficult to overcome. The construction of an efficient probe with high sensitivity, working at three different but close to each other frequencies, gives a possibility to obtain very interesting data on molecular dynamics in the heteronuclear systems [3–6, 10]. A uniquely designed and constructed probe of NMR spectrometer contains two independently tuned and electrically isolated coils installed in the coaxial position on the dewar. Three slightly differing frequencies on and off-resonance for protons and the frequency of 28.411 MHz for fluorine nuclei with no electrical interaction with one another can be produced.

This work puts forward an innovative and useful NMR off-resonance technique, in which detected proton ^1^H spins are spin-locked in the effective field $\vec{B}_{e}$ while fluorine ^19^F spins are continuously saturated for a long time. The saturation of fluorine spins for a long time simplifies the procedure for the analysis of experimental data, on account of the suppression of the heteronuclear ^1^H-^19^F dipolar coupling. For the analysis of NMR off-resonance data a model-free approach is applied. It is based on a rigorous theory advanced by Lipari and Szabo [1, 2] and is advantageous because it allows us to directly receive valuable parameters with well-defined physical significance [13–17]. According to this approach, molecular motion can be specified by set molecular parameters: a generalized order parameter *S*^2^and an effective correlation time *τ*_*e*_. The order parameter *S*^2^ measures the spatial restriction of the internuclear vector motions and describes the amplitude of molecular motion. Its values are in the range from 0 to 1. When its value is equal to 0, it corresponds to isotropic motion; the value 1 of parameter *S*^*2*^ means that the motion is completely restricted. The effective correlation time *τ*_*e*_ is a measure of the rate of motion and it is linked with a single correlation time *τ*_*M*_, describing the overall motion, by the equation:

1 $$\frac{1}{{\tau_{i} }} = \frac{1}{{\tau_{M} }} + \frac{1}{{\tau_{e} }} ,$$

where *τ*_*i*_ is the internal correlation time.

The object of this study is a solid state sample of guanidinium tetrafluoroborate [C(NH2)3]^+^BF4^-^ (GHBF4) [18]. The work focuses on research methodology. The sample is used as a model in order to demonstrate that the test results agree with the previous results [10]. Experimental data was obtained using cross-relaxation solid state NMR off-resonance technique and were analyzed using the Lipari-Szabo model-free formalism. From the dependence of the ratio of the cross-relaxation rate *σ*_*ρ*_ to the proton relaxation rate *ρ*_*ρ*_^*I*^ on the effective frequency *ω*_*e*_, three motion parameters: the order parameter *S*^2^, the correlation time τ_M_ and the internal correlation time τ_i_ were determined. It is a new application of the Lipari-Szabo model-free analysis to the interpretation of NMR off-resonance data on molecular dynamics in heteronuclear systems.

## Theory

Let us consider a system consisting of two spins *I* = 1/2 and *S* = 1/2 with magnetogyric ratios γ_I_ and γ_S_, respectively. In our case spin *I* refers to protons and *S* refers to fluorines. These two Zeeman reservoirs are connected to the lattice and to each other. Heteronuclear dipolar interaction mediates cross-relaxation between these Zeeman reservoirs and is characterized by the cross-relaxation rate *σ*. Both heteronuclear and homonuclear dipolar interactions mediate the spin–lattice relaxation process of each spin system separately with the proton and the fluorine spin–lattice relaxation rates *ρ*_I_ and *ρ*_S_, respectively. The equations describing the time evolution of the longitudinal spin polarizations: 〈*I*_*z*_〉(*t*) and 〈*S*_*z*_〉(*t*) in the laboratory frame were led out by Solomon [19]. The macroscopic differential equation for the longitudinal rotating frame relaxation of spin *I* modulated by dipolar interaction with spin *S* was derived by Peng and co authors [8]. The decay of the polarizations 〈*I*_*z*_’〉(*t*) along the effective field $\vec{B}_{e}$ is given by the following equation [8, 10]:

2 $$\frac{{d\left\langle {I_{z}^{'} } \right\rangle \left( t \right)}}{dt} = - \rho_{\rho }^{I} \left( {\left\langle {I_{z}^{'} } \right\rangle \left( t \right) - I^{'} \left( \infty \right)} \right) - \frac{{N_{I} }}{{N_{S} }}\sigma_{\rho }^{{}} \left( {\left\langle {S_{z} } \right\rangle \left( t \right) - S\left( \infty \right)} \right)$$

where *ρ*_*ρ*_^*I*^ is the off-resonance spin lattice relaxation rate in the rotating frame, *σ*_*ρ*_ is the cross-relaxation rate, *I*’(∞) is the equilibrium polarization for the *I* spins in the rotating frame, *S*(∞) is the polarization of the *S* spins at thermal equilibrium in the laboratory frame projected on the direction of the effective field $\vec{B}_{e}$; *N*_*I*_ and *N*_*S*_ describe the number of nuclei *I* and *S*, respectively.

When the *S* spins are saturated for a long time, the equation 〈*S*_*z*_〉(*t*) = 0 is satisfied and Eq. (2) can be rewritten as:

3 $$\frac{{d\left\langle {I_{z}^{'} } \right\rangle \left( t \right)}}{dt} = - \rho_{\rho }^{I} \left( {\left\langle {I_{z}^{'} } \right\rangle \left( t \right) - I^{'} \left( \infty \right)} \right) + \frac{{N_{I} }}{{N_{S} }}\sigma_{\rho } S\left( \infty \right) .$$

The analytical solution to Eq. (3) is monoexponential [9, 10] and is presented by the following equation:

4 $$\left\langle {I_{z}^{'} } \right\rangle \left( t \right) - I_{{}}^{'} \left( \infty \right) = \frac{{N_{I} }}{{N_{S} }}\frac{{\sigma_{\rho } }}{{\rho_{\rho }^{I} }}I\left( \infty \right)\frac{{\gamma_{S} }}{{\gamma_{I} }} + \left( {\left\langle {I_{z}^{'} } \right\rangle \left( 0 \right) - I^{'} \left( \infty \right) - \frac{{N_{I} }}{{N_{S} }}\frac{{\sigma_{\rho } }}{{\rho_{\rho }^{I} }}I\left( \infty \right)\frac{{\gamma_{S} }}{{\gamma_{I} }}} \right)\exp \left( { - \rho_{\rho }^{I} \cdot t} \right) ,$$

where 〈*I*_*z*_’〉(0) is the polarization of spins *I* at time *t* = 0 dependent on the initial conditions of the experiment.

Taking into account the following dependences:

5 $$\langle I_{z} '\rangle \left( t \right)\sim M'\left( t \right),\langle I'\left( \infty \right)\rangle \sim M_{\rho } ',\langle I\left( \infty \right)\rangle \sim M'\left( \infty \right),\langle I_{z} '\rangle \left( 0 \right)\sim M'\left( 0 \right)$$

Equation (4) can be rewritten to form:

6 $$M^{'} \left( t \right) - M_{\rho }^{'} = \frac{{N_{I} }}{{N_{S} }}\frac{{\sigma_{\rho }^{{}} }}{{\rho_{\rho }^{I} }}M^{'} \left( \infty \right)\frac{{\gamma_{S} }}{{\gamma_{I} }} + \left( {M^{'} \left( 0 \right) - M_{\rho }^{'} - \frac{{N_{I} }}{{N_{S} }}\frac{{\sigma_{\rho } }}{{\rho_{\rho }^{I} }}M'\left( \infty \right)\frac{{\gamma_{S} }}{{\gamma_{I} }}} \right)\exp \left( { - \rho_{\rho }^{I} \cdot t} \right)$$

In the NMR off-resonance experiment in the rotating frame, the effective magnetic field $\vec{B}_{e}$ is given by the sum of the vector $\varDelta \vec{B}$, parallel to the static field $\vec{B}_{0}$, and perpendicular vector $\vec{B}_{1}$, which represents the amplitude of the radio-frequency (rf) field. The $\varDelta \vec{B}$ component has the amplitude proportional to the difference between the resonance angular frequency *ω*_0_ and the angular frequency *ω* of field *B*_*1*_. The effective field $\vec{B}_{e}$ forms an angle *β* with the static magnetic field $\vec{B}_{0}$ and is defined by the equation:

7 $$\beta = \arctan \left( {\frac{{B_{1} }}{\varDelta B}} \right) .$$

**Fig. 1:**
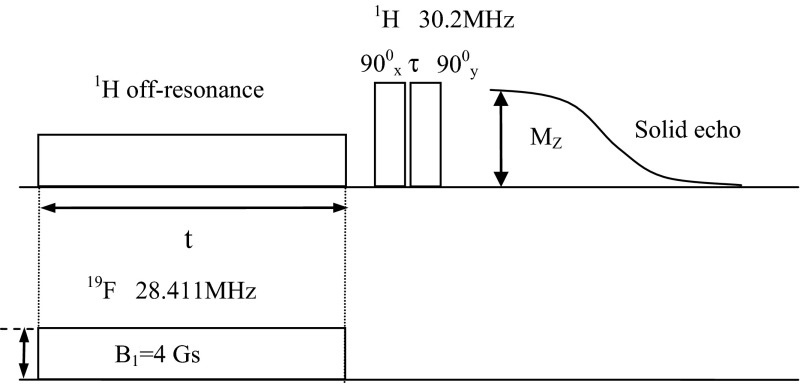
Pulse sequence for NMR off-resonance experiment in the rotating frame with the saturating of fluorines

In the NMR off-resonance experiment in the rotating frame with the saturating of fluorines (Fig. 1) after the completion of the off-resonance rf pulse, the parallel and the perpendicular components of magnetization *M*’(*t*), recover to the thermodynamic equilibrium in the static magnetic field *B*_0_. After the time *t*_*d*_ (about 200 μs for solid state), much longer than spin–spin relaxation time *T*_*2*_, the perpendicular component of magnetization disappears and then the second 90° rf on-resonance pulse or a $90^\circ_{x} - 90^\circ_{y}$ “solid echo” sequence on resonance is applied.Taking into account the following equations [10]:

8 $$M'\left( \infty \right) = M\left( \infty \right) { \cos } \beta ,M'\left( t \right) = M_{z} \left( t \right)/ { \cos } \beta ,M_{\rho } ' = M_{\rho } / { \cos } \beta ,$$

and introducing these expressions into Eq. (6) we get:

9 $$\frac{{M_{z} \left( t \right) - M_{\rho } }}{{M\left( \infty \right)\cos^{2} \beta }} = \frac{{N_{I} }}{{N_{S} }}\frac{{\sigma_{\rho } }}{{\rho_{\rho }^{I} }}\frac{{\gamma_{S} }}{{\gamma_{I} }} + \left( {\frac{M'\left( 0 \right)}{M\left( \infty \right)\cos \beta } - \frac{{M_{\rho } }}{{M\left( \infty \right)\cos^{2} \beta }} - \frac{{N_{I} }}{{N_{S} }}\frac{{\sigma_{\rho } }}{{\rho_{\rho }^{I} }}\frac{{\gamma_{S} }}{{\gamma_{I} }}} \right)\exp \left( { - \rho_{\rho }^{I} \cdot t} \right) .$$

In Eq. (9) the magnetization *M’*(0) of spins *I* at time *t* = 0 depends on the initial conditions of the experiment. According to the initial conditions of the experiment for *t* = 0 the following equation is satisfied:

10 $$M'\left( 0 \right) = M\left( \infty \right)\cos \beta ,$$

and Eq. (9) assumes the form:

11 $$\frac{{M_{Z} \left( t \right) - M_{\rho } }}{{M\left( \infty \right)\cos^{2} \beta }} = \frac{{N_{I} }}{{N_{S} }}\frac{{\sigma_{\rho } }}{{\rho_{\rho }^{I} }}\frac{{\gamma_{S} }}{{\gamma_{I} }} + \left( {1 - \frac{{M_{\rho } }}{{M\left( \infty \right)\cos^{2} \beta }} - \frac{{N_{I} }}{{N_{S} }}\frac{{\sigma_{\rho } }}{{\rho_{\rho }^{I} }}\frac{{\gamma_{S} }}{{\gamma_{I} }}} \right)\exp \left( { - \rho_{\rho }^{I} t} \right) ,$$

The monoexponential Eq. (11) was used to fit the experimental data, where: *N*_*I*_ and *N*_*S*_ are the numbers of proton and fluorine spins, *ρ*_*ρ*_^*I*^ is the proton spin–lattice relaxation rate in the rotating frame, *σ*_*ρ*_ is the cross-relaxation rate in the rotating frame.

By measuring the amplitude of NMR signal *M*_*z*_(*t*) as a function of duration *t* with the saturating of fluorines we are able to obtain the proton relaxation rate ρ_I_ and the ratio of the cross-relaxation rate *σ*_*ρ*_ to the proton relaxation rate $\rho_{\rho }^{I}$ by fitting formula (11) to the experimental data.

The $\frac{{\sigma_{\rho } }}{{\rho_{\rho }^{I} }}$ ratio contains information about nuclear Overhauser effect in the off-resonance rotating frame *NOE*_*ρ*_ and can be expressed by a suitable spectral density function:

12 $$\frac{{\sigma_{\rho } }}{{\rho_{\rho }^{I} }} = \frac{{ - J\left( {\omega_{I} - \omega_{S} - \omega_{e} } \right) + 6J\left( {\omega_{I} + \omega_{S} } \right)}}{{2\sin^{2} \left( \beta \right)J\left( {\omega_{e} } \right) + J\left( {\omega_{I} - \omega_{S} - \omega_{e} } \right) + 3J\left( {\omega_{I} } \right) + 6J\left( {\omega_{I} + \omega_{S} } \right)}} .$$

The NMR off-resonance data were analyzed using the Lipari-Szabo model-free approach, which assumes that the dipolar spectral density can be written as:

13 $$J\left( \omega \right) = \frac{2}{5}\left[ {\frac{{S^{2} \tau_{M} }}{{1 + \omega^{2} \tau_{M}^{2} }} + \frac{{\left( {1 - S^{2} } \right)\tau_{i} }}{{1 + \omega^{2} \tau_{i}^{2} }}} \right] ,$$

where

14 $$\frac{1}{{\tau_{i} }} = \frac{1}{{\tau_{M} }} + \frac{1}{{\tau_{e} }} ,$$

*S*^2^ is the order parameter, which is a measure of the restriction of the motion,

*τ*_*M*_ is the long rotational correlation time describing the global rotation of the molecule,

*τ*_*e*_ is the effective correlation time,

*τ*_*i*_ is the short internal correlation time, describing the fast reorientation of the local group.

Introducing Eq. (13) into Eq. (12) and assuming that:

$$\left( {\omega_{I} - \omega_{S} - \omega_{e} } \right)\tau_{M} \approx 1, \, \left( {\omega_{I} - \omega_{S} - \omega_{e} } \right)\tau_{i} < < 1, \, \left( {\omega_{I} + \omega_{S} } \right)\tau_{i} \approx 1, \, \left( {\omega_{I} + \omega_{S} } \right)\tau_{M} > > 1,\omega_{e} \tau_{M} \approx 1,\omega_{e} \tau_{i} < < 1,\omega_{I} \tau_{M} > > 1,\omega_{I} \tau_{i} \approx 1,$$

after a little algebra one gets:

15 $$\frac{{\sigma_{\rho } }}{{\rho_{\rho }^{I} }} = \frac{{ - \frac{2}{5}\left( {\frac{{\tau_{M} }}{{1 + \left( {\omega_{I} - \omega_{S} - \omega_{e} } \right)^{2} \tau_{M}^{2} }}} \right) + \frac{12}{5}\left( {\frac{1}{{\left( {\omega_{I} + \omega_{S} } \right)^{2} \tau_{M} }}} \right) + \left( {\frac{{1 - S^{2} }}{{S^{2} }}} \right)\tau_{i} A\left( {\tau_{i} } \right)}}{{\frac{4}{5}\sin^{2} \beta \left( {\frac{{\tau_{M} }}{{1 + \omega_{e}^{2} \tau_{M}^{2} }}} \right) + \frac{2}{5}\left( {\frac{{\tau_{M} }}{{1 + \left( {\omega_{I} - \omega_{S} - \omega_{e} } \right)^{2} \tau_{M}^{2} }}} \right) + \frac{1}{{\tau_{M} }}B + \left( {\frac{{1 - S^{2} }}{{S^{2} }}} \right)\tau_{i} C\left( {\tau_{i} } \right)}}$$

where

16 $$A\left( {\tau_{i} } \right) = - \frac{2}{5} + \frac{12}{5}\left( {\frac{1}{{1 + \left( {\omega_{I} + \omega_{S} } \right)^{2} \tau_{i}^{2} }}} \right) ,$$

17 $$C\left( {\tau_{i} } \right) = \frac{4}{5}\sin^{2} \beta + \frac{2}{5} + \frac{6}{5}\left( {\frac{1}{{1 + \omega_{I}^{2} \tau_{i}^{2} }}} \right) + \frac{12}{5}\left( {\frac{1}{{1 + \left( {\omega_{I} + \omega_{S} } \right)^{2} \tau_{i}^{2} }}} \right) ,$$

18 $$B = \frac{6}{5}\frac{1}{{\omega_{I}^{2} }} + \frac{12}{5}\frac{1}{{\left( {\omega_{I} + \omega_{S} } \right)^{2} }}\,{\text{is a characteristic constant for the spectrometer}}.$$

Equation (15) takes three independent parameters:

19 $$\begin{aligned} a = \tau_{M} , \hfill \\ b = \left( {\frac{{1 - S^{2} }}{{S^{2} }}} \right)\tau_{i} A\left( {\tau_{i} } \right), \hfill \\ c = \left( {\frac{{1 - S^{2} }}{{S^{2} }}} \right)\tau_{i} C\left( {\tau_{i} } \right). \hfill \\ \end{aligned}$$

From the ratio:

20 $$\frac{b}{c} = \frac{{A\left( {\tau_{i} } \right)}}{{C\left( {\tau_{i} } \right)}} = \frac{{ - \frac{2}{5} + \frac{12}{5}\left( {\frac{1}{{1 + \left( {\omega_{I} + \omega_{S} } \right)^{2} \tau_{i}^{2} }}} \right)}}{{\frac{4}{5}\sin^{2} \beta + \frac{2}{5} + \frac{6}{5}\left( {\frac{1}{{1 + \omega_{I}^{2} \tau_{i}^{2} }}} \right) + \frac{12}{5}\left( {\frac{1}{{1 + \left( {\omega_{I} + \omega_{S} } \right)^{2} \tau_{i}^{2} }}} \right)}} ,$$

the correlation time *τ*_*i*_ can be determined.

And then from Eq. (19) we are able to estimated the value of the order parameter *S*^*2*^.

## Experiment and results

**Fig. 2:**
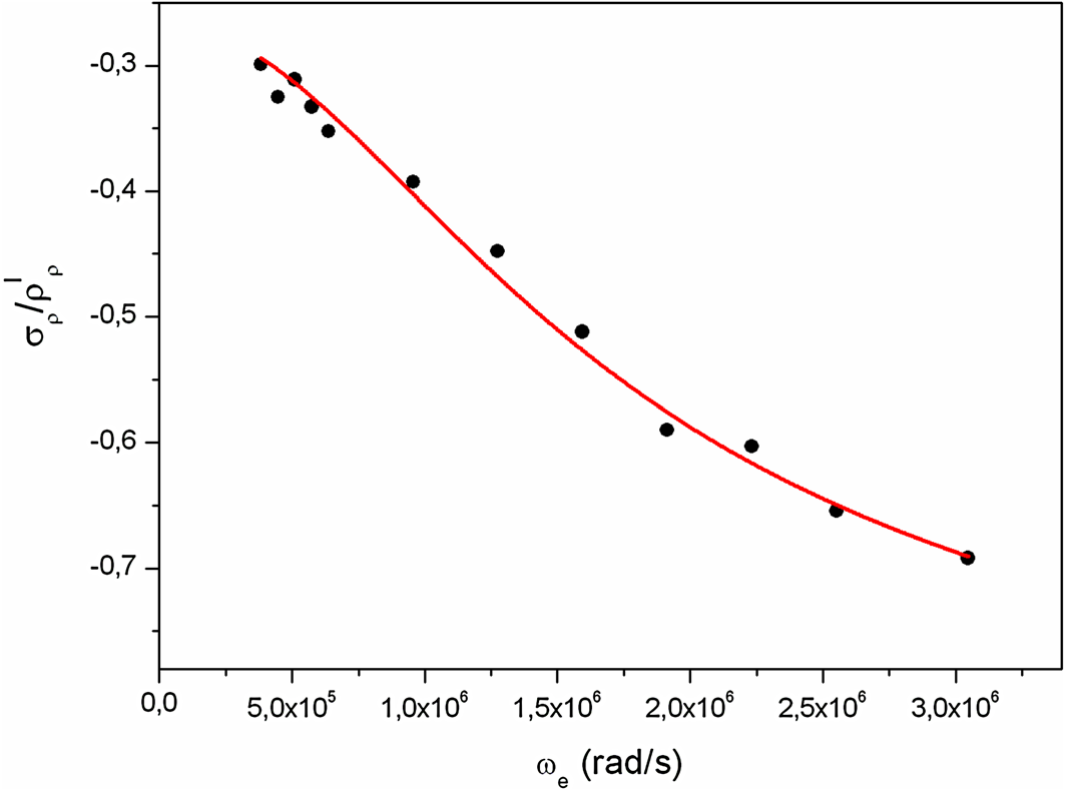
Dependence of *σ*_*ρ*_/*ρ*_*ρ*_^*I*^ratio as a function of the effective frequency *ω*_*e*_ in the rotating frame

The NMR off-resonance experiment in the rotating frame with the saturating of fluorines was performed on a solid state sample of guanidinium tetrafluoroborate [C(NH2)3]^+^BF4^−^ (GHBF4), synthesized in the Division of Molecular Crystals [18] at temperature 173 K on a homebuilt pulse spectrometer with a 10 % error [6]. A specially designed and constructed probe of NMR spectrometer contains two independently tuned and electrically isolated coils installed in the coaxial position on the dewar [3–6, 10]. Figure 1 presents a pulse sequence used in NMR off-resonance experiment. Two rf pulses are applied at the same time: one at the frequency off-resonance for the proton spins *I*, which produces the effective field $\vec{B}_{e}$ and the second rf pulse at the frequency of 28.411 MHz, which saturates the fluorine spin *S*. The fluorines are saturated throughout the whole duration of the off-resonance rf pulse. After the $90_{x}^\circ - \tau - 90_{y}^\circ$ pulse sequence at the resonance frequency for protons of 30.2 MHz, the solid echo is obtained. The echo amplitude is proportional to the proton magnetization *M*_*z*_(*t*) and depends on the duration *t* of the the off-resonance rf pulse. By fitting Eq (11) to the experimental data, the proton relaxation rate *ρ*_*ρ*_^*I*^ and the ratio of the cross-relaxation rate *σ*_*ρ*_ to the proton relaxation rate *ρ*_*ρ*_^*I*^ were estimated. Measurements were performed as a function of the intensity of the effective field $\vec{B}_{e}$ at the constant angle *β* equal to 10° by simultaneous changes in both the amplitude of rf field *B*_*1*_ and the angular frequency *ω* of field *B*_*1*_. The dependence of *σ*_*ρ*_/*ρ*_*ρ*_^*I*^ ratio as a function of the effective frequency *ω*_*e*_ in the rotating frame is presented in Fig. 2. The model-free approach was used to analyze experimental results. Equation (15) was used to fit experimental data. Parameters *a, b* and *c*, described by Eq. (19) were obtained by the method of least squares. Then the correlation time *τ*_*i*_ was estimated based on Eq. (20) and subsequently the order parameter *S*^*2*^.

The calculated internal correlation time *τ*_*i*_ is equal to 0.44 × 10^−8^ s, the long rotational correlation time describing the global rotation of the molecule *τ*_*M*_ = 1.04 × 10^−6^ s and *S*^*2*^ is equal to 0.4.

## Conclusion

The cross-relaxation NMR off-resonance experiment and the application of the Lipari-Szabo spectral density function make it possible to extract numerical values of the dimensionless order parameter *S*^2^ and of two correlation times, which describe two molecular processes: one with the overall correlation times *τ*_*M*_ and the other with the internal correlation time *τ*_*i*_.

Using the model-free approach allows us to study the molecular dynamics without the assumption of a specific model for internal motion.

In general, the measurements can be performed (1) at a constant angle *β* and variable effective frequency *ω*_e_, which enables us to separate relaxation mechanisms, (2) at a constant effective frequency *ω*_e_ and variable angle *β*.
